# Novel HDGF/HIF-1α/VEGF axis in oral cancer impacts disease prognosis

**DOI:** 10.1186/s12885-019-6229-5

**Published:** 2019-11-11

**Authors:** Yu-Wei Lin, Shih-Tsung Huang, Jian-Ching Wu, Tian-Huei Chu, Shih-Chung Huang, Ching-Chih Lee, Ming-Hong Tai

**Affiliations:** 1 Department of Radiation Oncology, Chi Mei Medical Center, Tainan City, 710 Taiwan; 2 Institute of Biomedical Science, National Sun Yat-sen University, Kaohsiung, 804 Taiwan; 3 Doctoral Degree Program in Marine Biotechnology, National Sun Yat-Sen University, Kaohsiung, 804 Taiwan; 4 Doctoral Degree Program in Marine Biotechnology, Academia Sinica, Taipei, 115 Taiwan; 5 Institute of Cellular and Organismic Biology, Academia Sinica, Taipei, 115 Taiwan; 6 Department of Pathology, Kaohsiung Chang Gung Memorial Hospital and Chang Gung University College of Medicine, Kaohsiung City, 833 Taiwan; 7 Center for Neuroscience, National Sun Yat-sen University, Kaohsiung, 804 Taiwan; 8 Department of Internal Medicine, Kaohsiung Armed Forces General Hospital, Kaohsiung, 802 Taiwan; 9 Department of Otolaryngology, Head and Neck Surgery, Kaohsiung Veterans General Hospital, Kaohsiung, 813 Taiwan; 10 School of Medicine, National Defense Medical Center, Taipei, 114 Taiwan; 11 Institute of Hospital and Health Care Administration, National Yang-Ming University, Taipei, 112 Taiwan; 12 Graduate Institute of Medicine, College of Medicine, Kaohsiung Medical University, Kaohsiung, 807 Taiwan

**Keywords:** Oral Cancer, Hepatoma-derived growth factor (HDGF), Vascular endothelial growth factor (VEGF), Hypoxia-inducible factor 1-α (HIF-1α), Nucleolin

## Abstract

**Background:**

Hepatoma-derived growth factor (HDGF) participates in angiogenesis and represents a negative prognostic factor in oral cancer. The current study was designed to elucidate the regulatory mechanism between HDGF and vascular endothelial growth factor (VEGF) and the clinical impact of oral cancer.

**Methods:**

TCGA data and surgical samples from oral cancer patients were used for the clinicopathological parameter and survival analysis. Human oral cancer SCC4 and SAS cells were treated with recombinant HDGF protein. VEGF gene expression and protein level were analyzed by RT-PCR, Western blotting, and enzyme-linked immunosorbent assay. The signaling pathways for regulating VEGF expression were investigated. The nucleolin neutralizing antibody and HIF-1α inhibitor were applied to SCC4 cells to investigate their effects on the HDGF-stimulated VEGF pathways.

**Results:**

TCGA and immunohistochemical analysis revealed a positive correlation between HDGF and VEGF expression in oral cancer tissues. Recombinant HDGF significantly increased VEGF gene and protein expression in oral cancer SCC4 cells in a dose-dependent manner. HDGF enhanced the phosphorylation levels of AKT and IkB and the protein level of HIF-1α and NF-κB. The nucleolin-neutralizing antibody abolished HDGF-stimulated HIF-1α, NF-κB and VEGF protein expression in SCC4 cells. The HIF-1α inhibitor antagonized the HDGF-induced VEGF gene expression. High VEGF expression was strongly correlated with HDGF expression, advanced disease, and poor survival.

**Conclusion:**

This study postulated a new pathway in which HDGF activated HIF-1α and then induced VEGF expression through binding to membrane nucleolin under normoxic conditions, leading to poor disease control. The HDGF/HIF-1α/VEGF axis is important for developing future therapeutic strategies.

## Background

Oral cancer is characterized by its aggressive behavior. Even after radical surgery followed by adjuvant radiotherapy and chemotherapy, the survival rate of oral cancer patients remains poor due to relentless recurrence or metastasis [1, 2].

Angiogenesis is required for tumor growth [3] and facilitates tumor recurrence and metastasis [4, 5] through perturbing the balance of proangiogenic and antiangiogenic factors. Among the proangiogenic factors, vascular endothelial growth factor (VEGF) is the most important one [6]. Angiogenesis plays a critical role in disease progression and mediates treatment resistance [7]. Therefore, understanding angiogenesis, particularly the VEGF pathway, is urgently needed for the risk stratification of oral cancer patients and the development of novel therapeutic targets.

Hepatoma-derived growth factor (HDGF) is a heparin-binding nuclear growth factor purified from the conditioned media of Huh-7 hepatoma cells [8–11]. HDGF overexpression has been found to correlate with advanced stages and poor prognosis in many types of cancer [12–17]. The possibility has been considered that HDGF induces angiogenesis [10, 18] through a direct effect or through the induction of VEGF release by regulating the VEGF upstream genes or VEGF promoters [19].

We have previously demonstrated that HDGF overexpression contributes to oncogenic processes and constitutes a novel negative prognostic factor for oral cancer [20]. HDGF expression has been hypothesized to play an important role in tumorigenesis and angiogenesis in oral cancer, which may be associated with the induction of angiogenic factors, leading to a more aggressive pattern of growth and poor prognosis [21]. However, the possible regulatory mechanism between HDGF and VEGF has not been explored.

Thus, the current study was designed to elucidate the possible interaction or regulatory mechanism between HDGF and VEGF and the possible clinical impact in oral cancer.

## Methods

### Reagents

Recombinant HDGF protein was generated as previously described [12]. The following reagents were purchased from Sigma-Aldrich (St. Louis, MO, USA): chetomin (C9623), Bay 11–7082 (B5556), Ponceau S solution (P7170), and β-actin (A5441). The following antibodies were purchased from Santa Cruz Biotechnology (Santa Cruz, CA, USA): VEGF (sc-152), p-AKT (sc-33,437), AKT (sc-1619), p-IκB (sc-8404), p65 (sc-372), STAT3 (sc-482) and the nucleolin neutralizing antibody (sc-8031). Other antibodies were obtained as follows: p-STAT3 (4113; Cell Signaling Technology, Inc., Danvers, MA, USA), IκB (ab32518; Abcam plc., Cambridge, UK), and HIF-1α (NB100–479; Novus International Inc., St Louis, MO, USA).

### Cell culture

Human tongue squamous carcinoma SCC4 (purchased from the Bioresource Collection and Research Center, Hsinchu, Taiwan) and SAS cells (purchased from Japanese Collection of Research Bioresources Cell Bank, Osaka, Japan) were 13th generations and cultured in DMEM/F12 (Invitrogen; Carlsbad, CA, USA) with 10% fetal bovine serum (FBS; HyClone, Logan, UT, USA), 2 mM glutamine, 100 U/ml penicillin (Invitrogen; Carlsbad, CA, USA) and 100 mg/ml streptomycin (Invitrogen; Carlsbad, CA, USA) at 37 °C in humidified air containing 5% CO_2_.

### Western blotting

Whole cell extracts were prepared and quantified by the Coomassie Plus Assay as previously described [22]. The PVDF membrane was blocked with 5% skim milk in TBS-T for 1 h and then incubated with the indicated primary antibodies and secondary antibodies conjugated with HRP (1:5000; Santa Cruz Inc.; Santa Cruz, CA, USA) for 1 h each. The signals on the membrane were detected using HRP chemiluminescent substrate (Millipore Corporation; Billerica, MA, USA) and exposed to X-ray film for signal detection.

### Quantitative real-time PCR

Total RNA purification and quantitative real-time PCR were performed as previously described [23]. The 2X SYBR Green PCR Master Mix (Thermo Fisher Scientific, Waltham, USA) and predesigned gene-specific primers for human VEGFA (NM_001025366.2) and β-actin (NM_007393.3) were used for quantitative real-time PCR. The data were normalized to β-actin and expressed as fold changes with respect to the control group. The primer sequences were as follows: VEGFA forward primer: 5′-**CCC TGA TGA GAT CGA GTA CA**-3′; VEGFA reverse primer: 5′-**AGG AAG CTC ATC TCT CCT AT**-3′; β-actin forward primer: 5′-**GGA ATC CTG TGG CAT CCA T**-3′; and β-actin reverse primer: 5′-**GCT CAG GAG GAG CAA TGA T**-3′.

### Enzyme-linked immunosorbent assay (ELISA)

The VEGF-A concentrations in the supernatants were determined by ELISA using a commercially available kit (Boster Biological Technology, Valley Ave, Pleasanton, CA). Briefly, after supernatant collection, the total cellular proteins were extracted and then measured by the bicinchoninic acid assay to assess the cell number in each group. The secreted VEGFA concentration was normalized to the total cellular protein level and is shown as the mean ± SD.

### Immunohistochemical staining and assessment

The surgically resected specimens from 102 oral cancer patients were included with approval from the institutional review board. Immunohistochemical analysis using the tissue microarray (TMA) consisting of surgically resected samples from oral cancer patients was performed as previously described [20] to delineate the correlation between HDGF expression, VEGF expression, and clinic-pathological parameters. Briefly, the slides were incubated with primary HDGF antibody (1:200 dilution) and VEGF antibodies (1:250; Santa Cruz; Santa Cruz, CA, USA) for 30 min and visualized using a peroxidase-conjugated secondary antibody, a polymer detection system (Zymed Laboratories, San Francisco, CA, USA) and 3,3-diaminobenzidine tetrahydrochloride (Sigma, St. Louis, MO). The sections were then counterstained with hematoxylin and eosin.

The percentage of tumor cells with definite moderate to intense nuclear or cytoplasmic immunoreactivity was scored, and the median of scores from multiple cores in the same patient was adopted as the labeling index (LI) for each marker, as previously described [12, 20, 24]. A total of 95 patient samples containing at least two preserved-tissue cores were scored and analyzed. Seven patients were excluded due to insufficient TMA samples. The cutoffs of the LIs to define high expression of HDGF were determined as follows: (1) high expression of nuclear HDGF (HDGF-N) if ≥40% of tumor nuclei were stained, (2) high expression of cytoplasmic HDGF (HDGF-C) if ≥40% of tumor cytoplasm was stained and (3) VEGF high expression if ≥50% of tumor cytoplasm was stained.

### Immunofluorescent staining of paraffin-embedded tissues

Immunofluorescence staining was performed on surgically resected specimens of oral cancer patients as described previously [25]. To investigate the expression of HDGF and VEGF, tissue sections were incubated with primary HDGF antibody (1:200 dilution) and VEGF antibodies (1:250; Santa Cruz; Santa Cruz, CA, USA). After wash step, tissue sections were incubated with appropriate fluorescent-labeled secondary antibodies then nuclei were stained with DAPI (Sigma-Aldrich, St. Louis, MO, USA). Finally, tissues were mounted with coverslips in fluorescence mounting medium (Dako corporation; Glostrup, Denmark). The fluorescent color of HDGF was Green (AlexaFluor488); VEGF was red (AlexaFluor546); nuclei were stained with blue color (DAPI). The microscope images were captured using Zeiss LSM 510 confocal imaging (200x magnification) and processed with ZEN 2 microscope image analysis software (Carl Zeiss; Jena, Germany).

### Computational biology analysis

HDGF and VEGF mRNA expression data were obtained from The Cancer Genome Atlas (TCGA). All the software and graphics for transcriptomics analysis were developed using in-house code implemented in MATLAB (MathWorks, Natick, MA, USA). HDGF and VEGF expression in the TCGA are reported as the fold changes between 1) oral cancer and healthy tissues and 2) head and neck cancer and healthy tissue. The correlation of HDGF and VEGFA mRNA expression in the TCGA dataset was analyzed by UCSC Xena (http://xena.ucsc.edu/).

### Statistical analysis

For the Western blotting, RT-PCR, and ELISA data, comparisons were performed by using one-way ANOVA followed by the Newman-Keuls post hoc test or t-test (for multiple comparisons) using Prism 5 (GraphPad Software, Inc., La Jolla, CA, USA). All the in vitro experiments in this study were triplicated. A probability value < 0.05 is considered to be statistically significant.

The associations among clinicopathologic factors, HDGF expression, and VEGF expression were evaluated using the X^2^ test, t-test, and ANOVA as appropriate. Estimates of disease-specific survival (DSS), metastasis-free survival (MFS), and local recurrence-free survival (LRFS) were calculated using the Kaplan–Meier method with log-rank test. The multivariable analyses of DSS, MFS, and LRFS were performed by using the Cox proportional hazards model with a stepwise approach. All tests were two-tailed, with a probability value < 0.05 considered to be statistically significant. Clinical statistical analyses were performed using SPSS 14 software (SPSS, Chicago, IL, USA).

## Results

### Correlation of HDGF and VEGF expression and clinicopathological parameters in oral cancer tissues

**Fig. 1:**
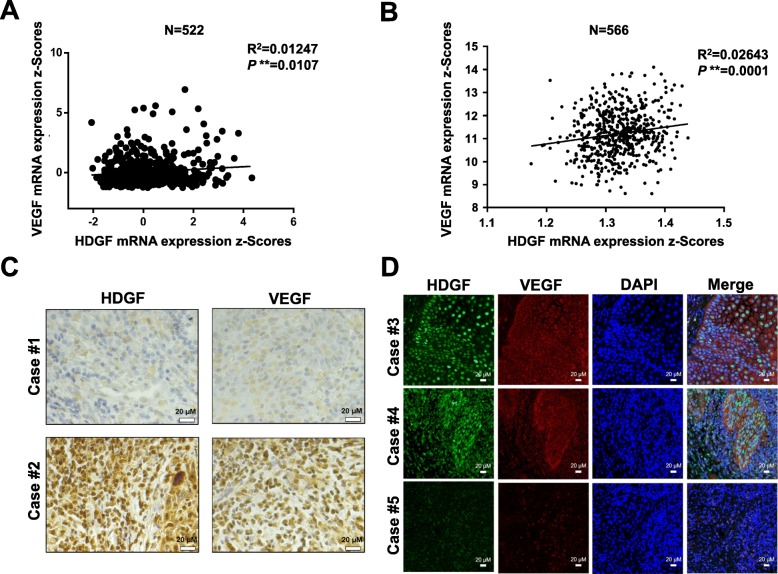
Correlation of HDGF and VEGF expression in oral cancer. **a**, **b** Correlation between HDGF and VEGF mRNA levels in oral cancer and head and neck cancer patients obtained by analysis of data from TCGA. HDGF expression is positively correlated with VEGFA expression in human head and neck squamous cell carcinoma tissues, including oral cancer. **c** Tissue microarray analysis of the correlation between HDGF and VEGF expression in oral cancer patients. The photographs were from two representative oral cancer patients. Case 1 (pT2N0M0, stage II) exhibited low-expression HDGF and VEGF immunostaining, whereas Case 2 (pT3N2M0, stage III) showed high-expression staining of both HDGF and VEGF. Scale bars, 20 μm. **d** Immunofluorescence staining of oral cancer patients. The fluorescent color of HDGF was Green (AlexaFluor 488); VEGF was red (AlexaFluor 546); nuclei were stained with blue color (DAPI). Case 3 (pT1N0M0, stage I) exhibited both high-intensity staining of HDGF and VEGF, whereas Case 4 (pT2N0M0, stage II) showed intermediate-intensity HDGF and VEGF immunofluorescence staining, and Case 5 (pT1N0M0, stage II) showed low-intensity staining of HDGF and VEGF. Scale bars, 20 μm

Because HDGF overexpression is correlated with angiogenesis and tumorigenesis, including in oral cancer [26], we investigated whether there was a relationship between HDGF and VEGF expression in oral cancer and head and neck cancer. According to TCGA data analysis (*n* = 522, oral cancer; *n* = 566, head and neck cancer [TCGA, Provisional cohort]), the HDGF and VEGF mRNA expression profile exhibited a strong positive correlation (*P* = 0.0107; R^2^ = 0.01247, oral cancer; *P* = 0.0001; R^2^ = 0.02643, head and neck cancer) (Fig. 1A and B). Therefore, these results suggested that HDGF expression was positively correlated with VEGF expression in human head and neck cancer and oral cancer. An immunohistochemistry assay revealed a positive correlation (*P* = 0.006) between HDGF-N expression and VEGF expression (Table 1). In addition, high expression of VEGF and HDGF-N were closely linked to advanced status of oral cancer, more advanced primary T stage and poorly differentiated histological grade. Higher VEGF expression also correlated with more advanced nodal status (*P* = 0.021). Immunohistochemical staining of HDGF and VEGF and immunofluorescence staining of oral cancer patients were shown in Fig. 1c and d.

**Table 1:** Correlation of HDGF, VEGF and clinicopathological data

Parameters		HDGF-N	*P* value	HDGF-C	*P* value	VEGF	*P* value
Gender	Male	50.0 ± 24.7	0.368	56.6 ± 25.1	0.650	67.0 ± 27.4	0.815
	Female	36.6 ± 40.4		63.3 ± 30.6		63.3 ± 15.3	
Age (years)		r = 0.126	0.223	r = 0.112	0.281	r = 0.04	0.970
Primary tumor (T)	T1-T2	41.2 ± 21.6	0.004*	53.6 ± 27.6	0.166	61.02 ± 27.9	0.038*
	T3	45.3 ± 24.1		54.7 ± 23.0		68.3 ± 28.4	
	T4	59.2 ± 27.1		61.5 ± 22.4		73.6 ± 24.8	
Nodal status (N)	N0	49.7 ± 22.6	0.924	54.6 ± 26.8	0.814	59.3 ± 23.6	0.021*
	N1	50.3 ± 20.2		64.7 ± 24.9		67.7 ± 27.9	
	N2	40.2 ± 28.9		55.9 ± 23.8		73.3 ± 28.3	
Extracapsular extension of metastatic nodes	Present	54.7 ± 29.6	0.149	58.7 ± 26.3	0.996	77.3 ± 25.5	0.052
	Absent	44.9 ± 24.7		58.7 ± 23.3		63.7 ± 29.4	
Histological grade	W-D	40.9 ± 25.1	0.013*	61.9 ± 24.3	0.061	58.1 ± 23.7	0.017*
	M-D	53.6 ± 22.9		57.6 ± 26.6		75.7 ± 24.4	
	P-D	59.1 ± 24.6		45.9 ± 21.9		68.8 ± 32.5	
Vascular invasion	Present	55.2 ± 27.1	0.205	57.1 ± 27.3	0.948	71.7 ± 25.9	0.325
	Absent	47.7 ± 24.3		56.7 ± 24.6		65.4 ± 27.4	
Perineurial invasion	Present	54.0 ± 27.6	0.290	52.9 ± 25.6	0.356	73.9 ± 23.6	0.127
	Absent	47.9 ± 24.1		58.3 ± 25.0		64.4 ± 27.9	
Tumor necrosis	Present	53.1 ± 25.7	0.276	66.4 ± 21.3	0.002*	70.4 ± 25.6	0.320
	Absent	47.3 ± 24.7		50.7 ± 25.7		64.7 ± 27.9	
CIS at adjacent mucosa	Present	55.2 ± 22.3	0.161	58.6 ± 24.1	0.658	66.9 ± 28.5	0.997
	Absent	47.2 ± 25.9		56.0 ± 25.7		66.9 ± 26.6	
HDGF-N				r = 0.131	0.204	r = 0.280	0.006*
HDGF-C						r = −0.022	0.831

*W-D*, Well differentiated; *M-D*, Moderately differentiated; *P-D*, Poorly differentiated; *CIS*, Carcinoma in situ; *HDGF-N*, Nuclear expression of hepatoma-derived growth factor; *HDGF-C*, Cytoplasmic expression of hepatoma-derived growth factor; *VEGF*, Vascular endothelial growth factor; *, Statistical significance; All values are presented as mean ± SD. The correlation analyses were evaluated using the *X*^*2*^ test, t-test, and ANOVA as appropriate

### Recombinant HDGF induced VEGF expression and release in oral cancer cells

**Fig. 2:**
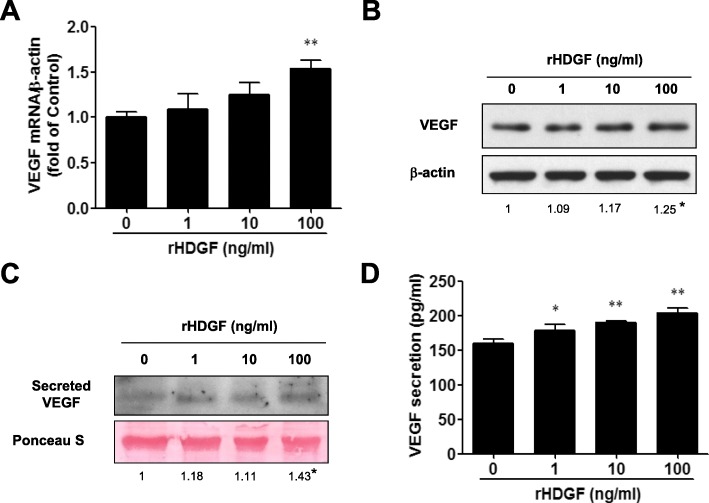
Effect of HDGF on VEGF expression in oral cancer cells. SCC4 cells were treated with the indicated concentration of recombinant HDGF protein for 24 h before harvest. **a** Relative gene expression levels of VEGF were analyzed by SYBR green-based RT-PCR. Data are expressed as the fold change with respect to the control group (means ± SD of triplicate experiments). **b** Cell lysates were analyzed using Western blotting, and the protein levels of VEGF/β-actin were measured and quantified. **c** The secreted VEGF protein levels in the supernatants were measured by Western blotting. Ponceau S staining was used as a loading control. **d** Levels of secreted VEGF protein (pg/ml) were detected by enzyme-linked immunosorbent assay (ELISA) in triplicate experiments. Data were mean of three experiments. *, *P* < 0.05; **, *P* < 0.01; ns, not statistically significant

To investigate whether HDGF regulated VEGF expression in oral cancer cells, SCC4 cells and SAS cells were treated with different concentrations of recombinant HDGF protein and then harvested for subsequent analysis. RT-PCR showed that exogenous HDGF protein significantly increased VEGF gene expression by approximately 1.5-fold compared with the control group in SCC4 cells (Fig. 2a, rHDGF 100 ng/ml, *P* < 0.01). Western blotting assays showed the protein levels of VEGF were also increased by HDGF stimuli in a dose-dependent manner (Fig. 2b and Additional file 1: Figure S1, rHDGF 100 ng/ml, *P* < 0.05). We next analyzed the secreted levels of VEGF by Western blotting and ELISA. As expected, more VEGF protein was secreted into the culture medium under HDGF stimulation than in the control group (Fig. 2c, rHDGF 100 ng/ml, P < 0.05). ELISA analysis revealed that HDGF enhanced a small but significant levels of VEGF secreted by SCC4 cells in a dose-dependent manner (Fig. 2d). Approximately additional 50 pg/ml VEGF was secreted in 100 ng/ml-rHDGF-treated group, comparing to the control group (Fig. 2d, *P* < 0.01). Therefore, these results supported that additional HDGF induced VEGF upregulation and expression in human oral cancer cells. SAS cells were treated with recombinant HDGF protein for 24 h before harvest. Western blotting showed the protein levels of VEGF was upregulated by HDGF stimulation in a dose-dependent manner (Additional file 1: Figure S2A-B).

### HDGF stimulates AKT/HIF-1α/NF-κB signaling in oral cancer cells

**Fig. 3:**
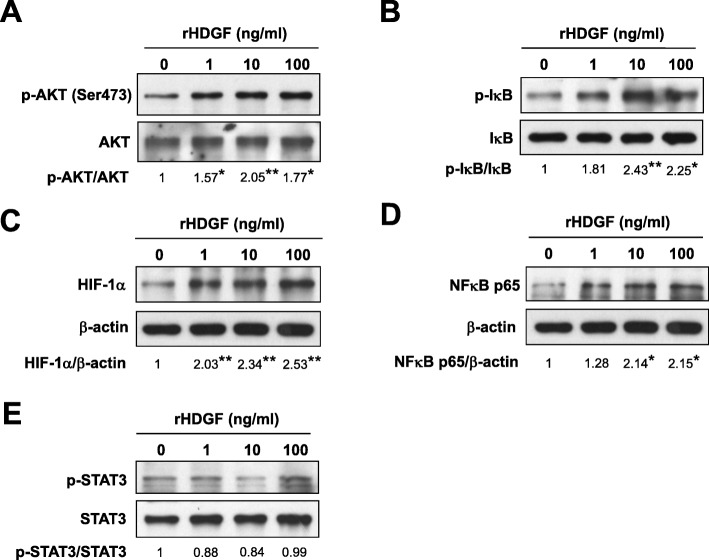
HDGF triggered AKT/HIF-1α/NF-κB signaling in SCC4 oral cancer cells. **a**-**d** Cells were treated with recombinant HDGF (1–100 ng/ml) for 24 h and then harvested for total protein extraction. The cell lysates were separated by SDS-PAGE and detected by Western blotting with the indicated primary antibodies. β-actin was used as an internal control for loading and transfer. Data were mean of three experiments. *, *P* < 0.05; **, *P* < 0.01; ns, not statistically significant

Given the well-known signaling pathways for regulating VEGF expression [27, 28], we then focused on the activation of specific transcription factors, including HIF-1α, NF-κB, and STAT3. SCC4 cells were treated with recombinant HDGF, and the levels of HIF-1α, NF-κB, and STAT3 were measured and quantified by Western blotting (Fig. 3a-d and Additional file 1: Figure S3A-D). HDGF enhanced the phosphorylation levels of AKT and IκB in the HDGF-treated group compared with the control group in SCC4 cells (Fig. 3a-b and Additional file 1: Figure S3A-B, rHDGF 10 ng/ml, *P* < 0.01). In addition, the protein levels of the transcriptional factors HIF-1α and NF-κB p65 were also upregulated under HDGF stimulation (HIF-1α, Fig. 3c and Additional file 1: Figure S3C, rHDGF 1 ng/ml, *P* < 0.01; NF-κB p65, Fig. 3d and Additional file 1: Figure S3D, rHDGF 10 ng/ml, *P* < 0.05), indicating that HDGF triggered the AKT/HIF-1α/NF-κB signaling pathway. HIF-1α was upregulated under HDGF stimulation in SAS cells (Additional file 1: Figure S2C, rHDGF 1 ng/ml, *P* < 0.01). However, HDGF treatment (even at a high dose of 100 ng/ml) did not affect the phosphorylation of STAT3, suggesting that HDGF did not elicit STAT3 activation in SCC4 cells (Fig. 3e and Additional file 1: Figure S3E). Together, these results implied that HDGF stimulated AKT/HIF-1α/NF-κB signaling, thereby modulating VEGF expression in oral cancer cells.

### Antibody neutralization of surface nucleolin abolished HDGF-stimulated AKT/HIF1α/NF-κB/VEGF signaling in oral cancer cells

**Fig. 4:**
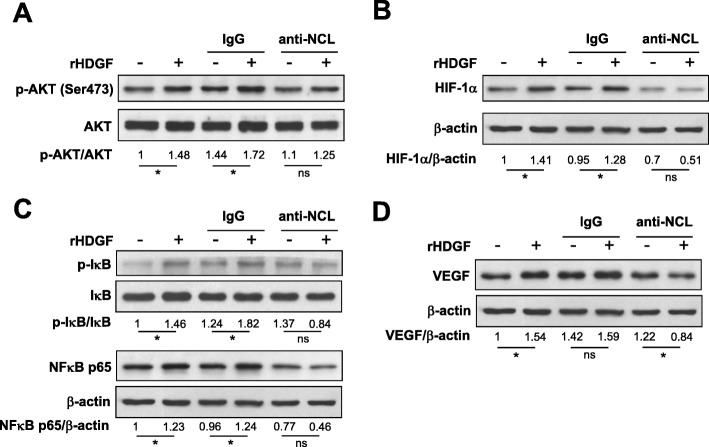
The Neutralizing antibody against nucleolin eliminate HDGF-stimulated AKT/HIF-1α/NF-κB/VEGF signaling in SCC4 oral cancer cells. **a**-**d** SCC4 cells were treated with recombinant HDGF protein (100 ng/ml) in the presence of anti-NCL or anti-IgG antibody (5 μg/ml) for 24 h before total protein extraction. Cell lysates were subjected to Western blotting with the indicated antibodies. β-actin was used as an internal control for loading and transfer. Data were mean of three experiments. *, *P* < 0.05; **, *P* < 0.01; ns, not statistically significant

Because the surface nucleolin/AKT axis has been found to participate in transmitting the oncogenic signaling of HDGF [22], we investigated whether blockage of the HDGF/nucleolin axis by antibody neutralization affected HDGF-stimulated HIF-1α, NF-κB and VEGF expression in SCC4 cells. Western blotting analysis demonstrated that the additional recombinant HDGF was unable to enhance phosphorylation levels of AKT and HIF-1α protein under the co-treatment with the neutralizing antibodies against nucleolin in SCC4 cells (Fig. 4a-b and Additional file 1: Figure S4A-B). Moreover, blocking the HDGF/nucleolin axis not only diminished the HDGF-stimulated phosphorylation of IκB and NF-κB p65 but also significantly reduced VEGF protein expression (Fig. 4c-d and Additional file 1: Figure S4C-E, *P* < 0.05). These results suggested that the nucleolin-mediated signaling pathway is important for HDGF-modulated VEGF expression.

### Application of the HIF-1α inhibitor chetomin antagonized HDGF-induced VEGF upregulation in oral cancer cells

**Fig. 5:**
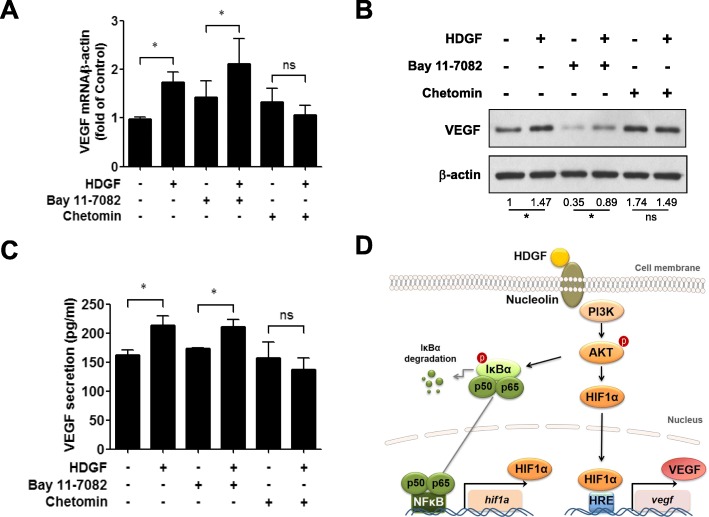
Effects of chetomin and Bay 11–7082 on HDGF-induced VEGF upregulation in SCC4 oral cancer cells. Cells were treated with recombinant HDGF protein (100 ng/ml) in the presence of Bay 11–7082 (10 nM) or chetomin (10 nM) for 24 h. **a** Relative gene expression levels of VEGF were analyzed by SYBR Green-based RT-PCR. Data are expressed as the fold change with respect to the control group (means ± SD of triplicate experiments). **b** The protein levels of VEGF were analyzed by Western blotting and normalized to β-actin expression. (**c**) the levels of secreted VEGF protein (pg/ml) were detected by ELISA in triplicate experiments. **d** Scheme for HDGF-regulated VEGF transcription in oral cancer cells. Data were mean of three experiments. *, *P* < 0.05; **, *P* < 0.01; ns, not statistically significant

To further investigate which of the transcription factors HIF-1α and NF-κB were dominant in HDGF-induced VEGF gene expression, we employed the HIF-1α inhibitor chetomin and the NF-κB inhibitor Bay 11–7082. RT-PCR analysis showed no significant difference in VEGF mRNA levels with or without additional HDGF in the chetomin group (Fig. 5a, P, not statistically significant). The application of chetomin potently suppressed the HDGF-induced VEGF gene expression. On the other hand, HDGF treatment was able to induce VEGF upregulation even in the presence of Bay 11–7082 (Fig. 5a). Western blot assays demonstrated that chetomin suppressed the VEGF protein expression induced by HDGF (Fig. 5b). Although Bay 11–7082 could inhibit the basal level of VEGF, VEGF was still enhanced in HDGF-treated cells (Fig. 5b). Moreover, ELISA also revealed that chetomin eliminated the increased secretion of VEGF protein induced by HDGF (Fig. 5c, P, not statistically significant). Thus, HIF-1α signaling plays a critical role in HDGF-induced VEGF gene regulation.

### Univariate log-rank analyses of survival

**Fig. 6:**
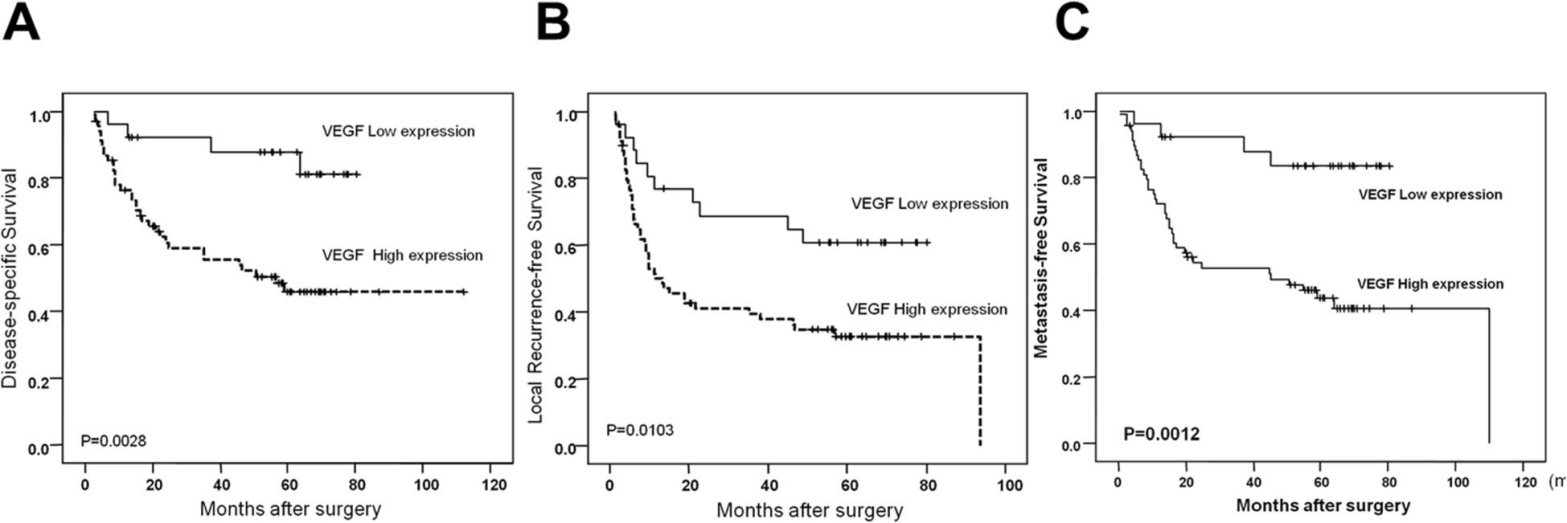
Survival and VEGF expression. The disease-specific (**a**), local recurrence-free (**b**), and distant metastasis-free (**c**) survival of patients with low expression and high expression of VEGF in oral cancer patients.

According to univariate survival analysis, postoperative concurrent chemoradiotherapy (Post-OP CCRT), histological grade, and high expression of HDGF-N and VEGF were statistically significant prognostic predictors for DSS, MFS, and LRFS. The univariate survival analysis is summarized in Additional file 1: Table S1. High VEGF expression predicted a higher rate of local and distant recurrence and shorter DSS in the Kaplan–Meier survival analysis (Fig. 6).

### Multivariate analyses of survival

In the multivariate comparison (Table 2), advanced primary T stage (*P* = 0.0001; RR, 5.98), higher histological grade (*P* = 0.0014; RR, 7.50), lack of Post-OP CCRT (*P* < 0.0001; RR, 6.89), high expression of HDGF-N (*P* = 0.028; RR, 3.04) and high expression of VEGF (*P* = 0.0183; RR, 4.09) represented independent negative prognostic factors for DSS. For MFS, strong independent prognostic factors were advanced primary T stage (*P* = 0.0003; RR, 4.39), higher histological grade (*P* = 0.0009; RR, 6.70), lack of Post-OP CCRT (P < 0.0001; RR, 5.61), and high expression of VEGF (*P* = 0.0153; RR, 4.01). Lack of Post-OP CCRT (*P* = 0.0117; RR, 2.00), high expression of VEGF (*P* = 0.0461; RR, 2.10) and HDGF-N (*P* = 0.0285; RR, 2.14) were predictive of inferior LRFS.

**Table 2:** Multivariate analyses of HDGF and VEGF

Variable		DSS			MFS			LRFS		
	Category	RR	95% CI	*p*-value	RR	95% CI	*p*-value	RR	95% CI	*p*-value
Primary tumor (T)	T1-T2	1	–	0.0001*	1	–	0.0003*			
	T3	1.11	0.40–3.12		1.09	0.41–2.88				
	T4	5.98	2.50–14.33		4.39	2.00–9.63				
Histological grade	W-D	1	–	0.0014*	1	–	0.0009*	1	–	0.0981
	M-D	3.09	1.11–8.62		3.15	1.20–8.23		1.247	0.62–2.48	
	P-D	7.50	2.48–22.67		6.70	2.50–19.60		2.109	1.04–4.27	
Nodal status (N)	N0	1	–	0.1073	1	–	0.2104			
	N1	2.31	0.90–5.95		1.77	0.76–4.10				
	N2	2.98	1.03–8.58		2.37	0.88–6.37				
Post-OP CCRT	Yes	1	–	< 0.0001*	1	–	< 0.0001*	1	–	0.0117*
	No	6.89	2.90–16.41		5.61	2.54–12.39		2.003	1.17–3.44	
VEGF	Low expression	1	–	0.0183*	1	–	0.0153*	1	–	0.0461*
	High expression	4.09	1.27–13.15		4.01	1.30–12.30		2.100	1.01–3.45	
HDGF-N	Low expression	1	–	0.0280*	1	–	0.0534	1	–	0.0285*
	High expression	3.04	1.13–8.22		2.36	0.99–5.62		2.141	1.08–4.23	

Post-OP CCRT, postoperative concurrent chemoradiotherapy; W-D, well differentiated; M-D, moderately differentiated; P-D, poorly differentiated; HDGF-N, nuclear expression of hepatoma-derived growth factor; VEGF, vascular endothelial growth factor; DSS, disease-specific survival; MFS, metastasis-free survival; LRFS, local recurrence-free survival; *, Statistical significance

## Discussion

Angiogenesis is essential for cancer progression, metastasis and treatment resistance. The regulation of angiogenesis involves a number of critical growth factors, cytokines, signaling cascades and cellular processes that are triggered in response to either a hypoxic or an inflammatory stimulus [29]. Hypoxia- and inflammation-driven angiogenesis are regulated via distinctly different and yet overlapping pathways [30].

Through correlation analysis of the immunohistochemistry assay and TCGA data, these results provided support for the interaction between HDGF and VEGF expression in oral cancer. In this study, for the first time, we demonstrated that HDGF enhanced VEGF expression in oral cancer cells at the mRNA level, protein level and secretion level with a dose-dependent manner.

The mechanism through which HDGF induces or regulates VEGF expression in tumor cells remains unclear. HDGF has been reported to stimulate the proliferation and invasion of hepatocellular carcinoma cells via PI3K/AKT signaling [22, 31]. Indeed, activation of the PI3K/AKT pathway in both tumor and endothelial cells increases VEGF secretion by both HIF-1α-dependent and HIF-1α-independent mechanisms [32–34].

In hypoxia-driven angiogenesis, hypoxia activates the PI3K/AKT pathway to prevent the posttranslational hydroxylation and the subsequent degradation of HIF-1α, allowing it to accumulate and then translocate to the nucleus, where it upregulates VEGF production pathways [29, 35–37]. The inflammatory stimulus activates the PI3K/AKT pathway, leading to the phosphorylation of IκBα. IκBα is degraded, allowing NF-κB subunits p50 and p65 to translocate into the nucleus and activate VEGF production [29, 38].

HIF-1 has been shown to essentially control the cellular response to hypoxia. Evidence has emerged that HIF-1α is also responsive to stimuli under normoxic conditions [39]. One important mechanism underlying these normoxic conditions is the transcriptional regulation of HIF-1α by NF-κB [40], which is the key promoter in the inflammatory angiogenic pathway [29, 39]. Recently, HIF-1α has been reported that directly bound to the HDGF promoter region, which was highly correlated with pancreatic cancer-associated fibrosis under normoxic condition [41].

Our data have shown that exogenous HDGF protein not only stimulated the phosphorylation levels of AKT and IκB but also increased the protein levels of the transcriptional factors HIF-1α and NF-κB p65 in oral cancer cells. The Western blotting results (Fig. 3) showed HDGF in a dose of 10 ng/ml was able to enhance more than two folds of phosphorylation levels of AKT and IκB; additional HDGF in the low-dose of 1 ng/ml could induce two folds of the protein levels of HIF-1α. The upregulation of phosphorylated IκB implied a loss of NF-κB blockage by IκB, leading to NF-κB activation and subsequently to modulated HIF-1α expression or VEGF production. This finding offered a rationale for how HDGF simultaneously triggered the AKT/HIF-1α and NF-κB signaling pathways in oral cancer cells.

HDGF has been demonstrated to bind directly to surface nucleolin (NCL) and activate the NCL/PI3K/AKT axis in hepatoma cells during liver carcinogenesis [22]. Here, we applied a neutralizing antibody against nucleolin that was able to abolish the HDGF-stimulated phosphorylation levels of AKT, IκB and NF-κB p65 and the HDGF-stimulated protein levels of HIF-1α VEGF. These results suggest that surface nucleolin plays a pivotal role in mediating the HDGF-induced AKT/HIF-1α signaling and NF-κB signaling pathways, ultimately modulating VEGF expression in oral cancer cells.

Studies have shown that the binding of both STAT3 and HIF-1α to the VEGF promoter is essential for the maximum transcription of VEGF mRNA under hypoxia [42]. STAT3 signaling is required for VEGF- and PI3K/AKT-mediated HIF-1α expression. Blocking STAT3 abolished both HIF-1 and VEGF expression [43]. However, whether STAT3 contributes to HIF-1 expression/activity independently of AKT remains to be determined. Here, HDGF did not modulate the phosphorylation levels of the transcription factor STAT3 even at a high dose (100 ng/ml), suggesting that STAT3 activation was not modulated by recombinant HDGF in SCC4 cells. This result implied that HDGF-stimulated VEGF expression might act through alternative AKT/HIF-1α and NF-κB signaling pathways but not the STAT3 pathway in oral cancer cells.

To confirm the signaling pathway between HDGF and VEGF, a HIF-1α inhibitor (chetomin) and an NF-κB inhibitor (Bay 11–7082) were used. Mild upregulation in VEGF mRNA level, protein level, and secreted protein level were noted in the chetomin alone group. In the cotreatment group of HDGF and chetomin, the VEGF levels were reduced without further enhancement that suggested chetomin was able to eliminate HDGF-induced VEGF expression pathway in SCC4 cells. In the other hand, Bay 11–7082 has some suppressive effects on the VEGF mRNA level, protein level, and secreted protein level. The HDGF cotreatment with Bay 11–7082 was able to upregulate the VEGF mRNA and protein levels even under the possible suppression caused by Bay 11–7802. Therefore, the current study revealed the pivotal role of HIF-1α signaling in the HDGF-mediated upregulation of VEGF (Fig. 5d). There are some limitations to this study. We only analyzed three common signal transduction pathways for the possible regulatory VEGF pathways by Western blotting. Western blotting can only identify a single protein-protein interaction but not for weak or transient interactions and evaluate numerous pathways will be time-consuming. However, the two cell lines in the current study showed consistent results. Also, we validated the correlation between HDGF and VEGF in the clinical data.

In the current study, the cohort of patients with oral cancer who received radical treatment was selected to assess the prognostic value of VEGF immunohistochemical staining. High expression of VEGF was strongly correlated with HDGF-N expression, primary T stage, nodal status and histological grade. In a previous study, high expression of HDGF seemed limited to locally aggressive behavior only [20]. Here, high expression of VEGF was associated with a greater likelihood of both local and distant recurrence. VEGF is capable of increasing vascular permeability in both blood and lymphatic vessels and helps cancer cells enter lymphatic or blood vessels and become established in both local lymph nodes and at distant sites [4, 44]. In multivariate analysis, high expression of VEGF was the most significant predictor for all survival endpoints (LRFS, DMS, and DSS).

Antiangiogenic agents can potentially modulate the tumor microenvironment and induce radiosensitivity and chemosensitivity. Using antiangiogenic agents alone or in combination with conventional therapies in oral cancer is a promising new approach [45]. In the current study, HDGF activated HIF-1α and then induced VEGF expression, leading to poor disease control. The combination of antiangiogenic agents and HIF-1 inhibitors might be efficacious, as antiangiogenic agents would cut off the tumor’s blood supply, and HIF-1α inhibitors could potentiate the effect of antiangiogenic agents and reduce the potential for the development of drug resistance [46]. Therefore, the HDGF/nucleolin/HIF-1α/VEGF axis is a very attractive target for oral cancer treatment.

## Conclusions

In summary, this study is the first to report the association between HDGF and VEGF and prognosis in oral cancer. Our study postulated a new pathway in which HDGF activated HIF-1α and the NF-κB signaling pathway and then increased VEGF expression through binding to membrane NCL under normoxic conditions. The HDGF/HIF-1α/VEGF axis is important for developing future therapeutic strategies.

## Supplementary information

**Additional file 1: Table S1. **Univariate log-rank analyses of HDGF and VEGF. **Figure S1. **Effect of HDGF on VEGF expression in oral cancer cells. **Figure S2.** Effect of HDGF on VEGF expression in oral cancer cells. **Figure S3.** HDGF triggered AKT/HIF-1α/NF-κB signaling in SCC4 oral cancer cells. **Figure S4.** The Neutralizing antibody against nucleolin eliminates HDGF-stimulated AKT/HIF-1α/NF-κB/VEGF signaling in SCC4 oral cancer cells.

## Data Availability

All data analyzed during this study are included in this published article.

## Abbreviations

DSS: Disease-specific survival
ELISA: Enzyme-linked immunosorbent assay
HDGF: Hepatoma-derived growth factor
HDGF-C: Cytoplasmic HDGF
HDGF-N: Nuclear HDGF
LRFS: Local recurrence-free survival
MFS: Metastasis-free survival
Post-OP CCRT: Postoperative concurrent chemoradiotherapy
TCGA: The Cancer Genome Atlas
TMA: Tissue microarray
VEGF: Vascular endothelial growth factor
