# Supplementary material for: Novel HDGF/HIF-1α/VEGF axis in oral cancer impacts disease prognosis
Source: BMC Cancer. 2019 Nov 11;19:1083. doi: 10.1186/s12885-019-6229-5 (PMC6849302; doi:10.1186/s12885-019-6229-5)
Supplement: Supplementary file 1 — Additional file 1: Table S1. Univariate log-rank analyses of HDGF and VEGF. Figure S1. Effect of HDGF on VEGF expression in oral cancer cells. Figure S2. Effect of HDGF on VEGF expression in oral cancer cells. Figure S3. HDGF triggered AKT/HIF-1α/NF-κB signaling in SCC4 oral cancer cells. Figure S4. The Neutralizing antibody against nucleolin eliminates HDGF-stimulated AKT/HIF-1α/NF-κB/VEGF signaling in SCC4 oral cancer cells. [file 12885_2019_6229_MOESM1_ESM.docx]

**Additional file 1**

**Table S1: Univariate log-rank analyses of HDGF and VEGF**

|  |  |  | **DSS** | | **MFS** | | **LRFS** | |
| --- | --- | --- | --- | --- | --- | --- | --- | --- |
| Parameters |  | **No. of case** | **No. of event** | **Sig.** | **No. of event** | **Sig.** | **No. of event** | **Sig.** |
| **Gender** | Male | 92 | 38 | 0.1955 | 43 | 0.1704 | 55 | 0.3693 |
|  | Female | 3 | 0 |  | 0 |  | 1 |  |
| **Age (years)** | <60 | 80 | 15 | 0.8602 | 38 | 0.6464 | 49 | 0.5542 |
|  | ≧60 | 15 | 5 |  | 5 |  | 7 |  |
| **Primary tumor (T)** | T1-T2 | 44 | 10 | **0.0137*** | 13 | **0.0369*** | 22 | 0.3309 |
|  | T3 | 15 | 8 |  | 8 |  | 9 |  |
|  | T4 | 36 | 20 |  | 22 |  | 25 |  |
| **Nodal status (N)** | N0 | 37 | 7 | **0.0018*** | 10 | **0.0033*** | 19 | 0.1476 |
|  | N1 | 15 | 8 |  | 8 |  | 9 |  |
|  | N2 | 43 | 23 |  | 25 |  | 28 |  |
| **Post-OP CCRT** | Yes | 50 | 15 | **0.0007*** | 18 | **0.0008*** | 27 | **0.0147*** |
|  | No | 45 | 23 |  | 25 |  | 29 |  |
| **Extracapsular extension of metastatic node** | Present | 31 | 17 | 0.1510 | 19 | 0.0663 | 20 | 0.2153 |
|  | Absent | 34 | 16 |  | 16 |  | 20 |  |
| **Histological grade** | W-D | 39 | 6 | **0.0004*** | 7 | **0.0003*** | 16 | **0.0365*** |
|  | M-D | 35 | 18 |  | 20 |  | 23 |  |
|  | P-D | 21 | 14 |  | 16 |  | 17 |  |
| **Vascular invasion** | Present | 24 | 11 | 0.5538 | 14 | 0.3561 | 17 | 0.5921 |
|  | Absent | 71 | 27 |  | 29 |  | 39 |  |
| **Perineurial invasion** | Present | 26 | 14 | 0.0713 | 15 | 0.3129 | 17 | 0.3824 |
|  | Absent | 69 | 24 |  | 28 |  | 39 |  |
| **Tumor necrosis** | Present | 37 | 15 | 0.9073 | 18 | 0.6118 | 32 | 0.3478 |
|  | Absent | 58 | 23 |  | 25 |  | 24 |  |
| **CIS at adjacent mucosa** | Present | 28 | 11 | 0.9154 | 13 | 0.8429 | 17 | 0.8814 |
|  | Absent | 67 | 27 |  | 30 |  | 39 |  |
| **Surgical margin** | Clear | 85 | 34 | 0.9362 | 39 | 0.8535 | 51 | 0.8932 |
|  | Unclear | 8 | 3 |  | 3 |  | 4 |  |
| **HDGF-N** | Low expression | 32 | 6 | **0.0069*** | 9 | **0.0168*** | 12 | **0.0047*** |
|  | High expression | 63 | 32 |  | 34 |  | 44 |  |
| **HDGF-C** | Low expression | 25 | 9 | 0.7027 | 11 | 0.7061 | 14 | 0.7204 |
|  | High expression | 70 | 29 |  | 32 |  | 42 |  |
| **VEGF** | Low expression | 26 | 4 | **0.0028*** | 4 | **0.0012*** | 10 | **0.0130*** |
|  | High expression | 69 | 34 |  | 39 |  | 46 |  |

Post-OP CCRT, postoperative concurrent chemoradiotherapy; W-D, well differentiated; M-D, moderately differentiated; P-D, poorly differentiated; CIS, carcinoma in situ; DSS, disease-specific survival; MFS, metastasis-free survival; LRFS, local recurrence-free survival.; *, Statistical significance

**
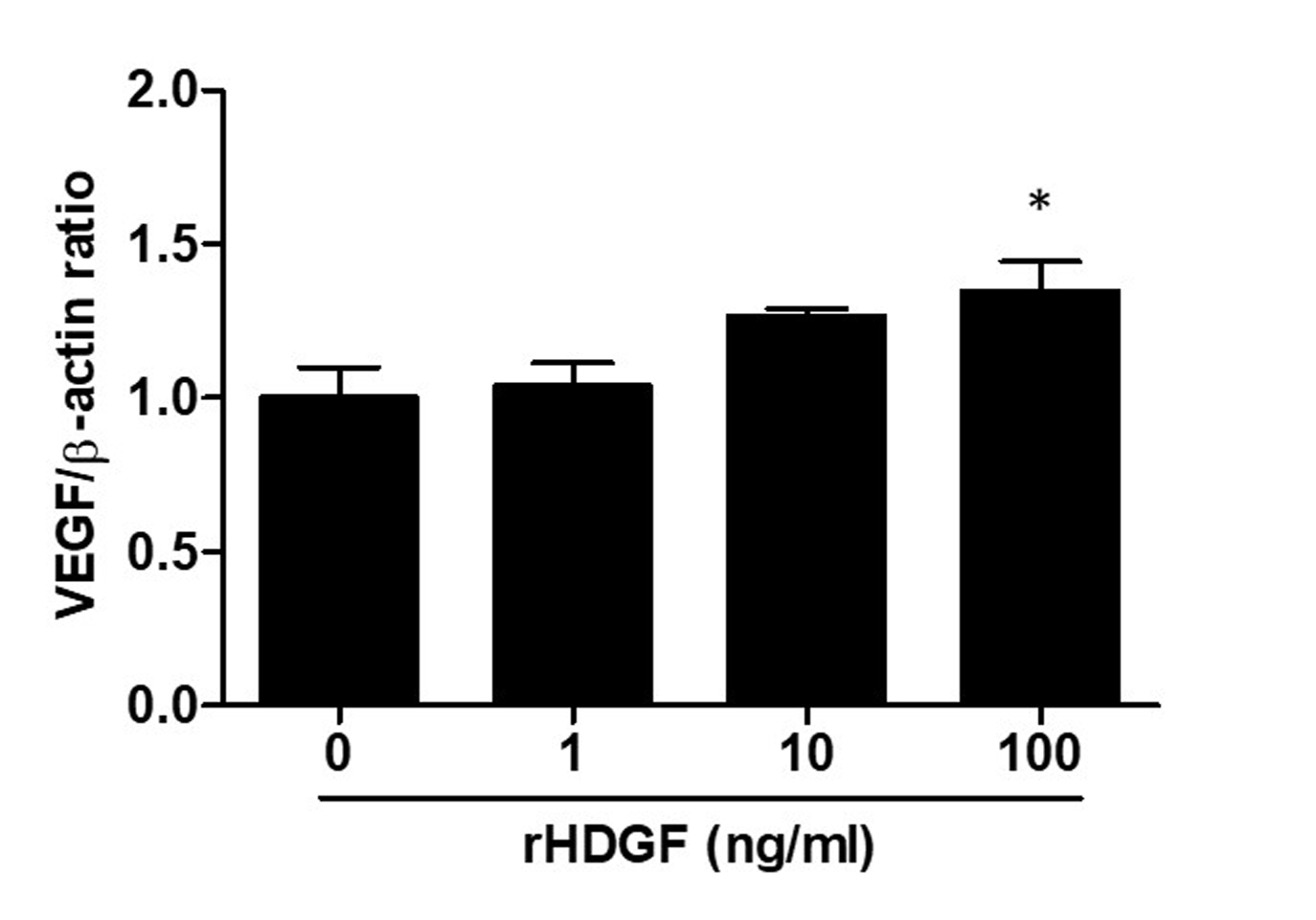
**

**Figure S1. Effect of HDGF on VEGF expression in oral cancer cells.** SCC4 cells were treated with the indicated concentration of recombinant HDGF protein for 24 h before harvest. Cell lysates were analyzed using Western blotting, and the protein levels of VEGF/β-actin were measured and quantified. Data were mean of three experiments. *, P<0.05; **, P<0.01; ns, not statistically significant.

**Figure S2. Effect of HDGF on VEGF expression in oral cancer cells.** (A-C) SAS cells were treated with the indicated concentration of recombinant HDGF protein for 24 h before harvest. Cell lysates were analyzed using Western blotting, and the protein levels of VEGF/β-actin and HIF-1α/β-actin were measured and quantified. Data were mean of three experiments. *, P<0.05; **, P<0.01; ns, not statistically significant.

**
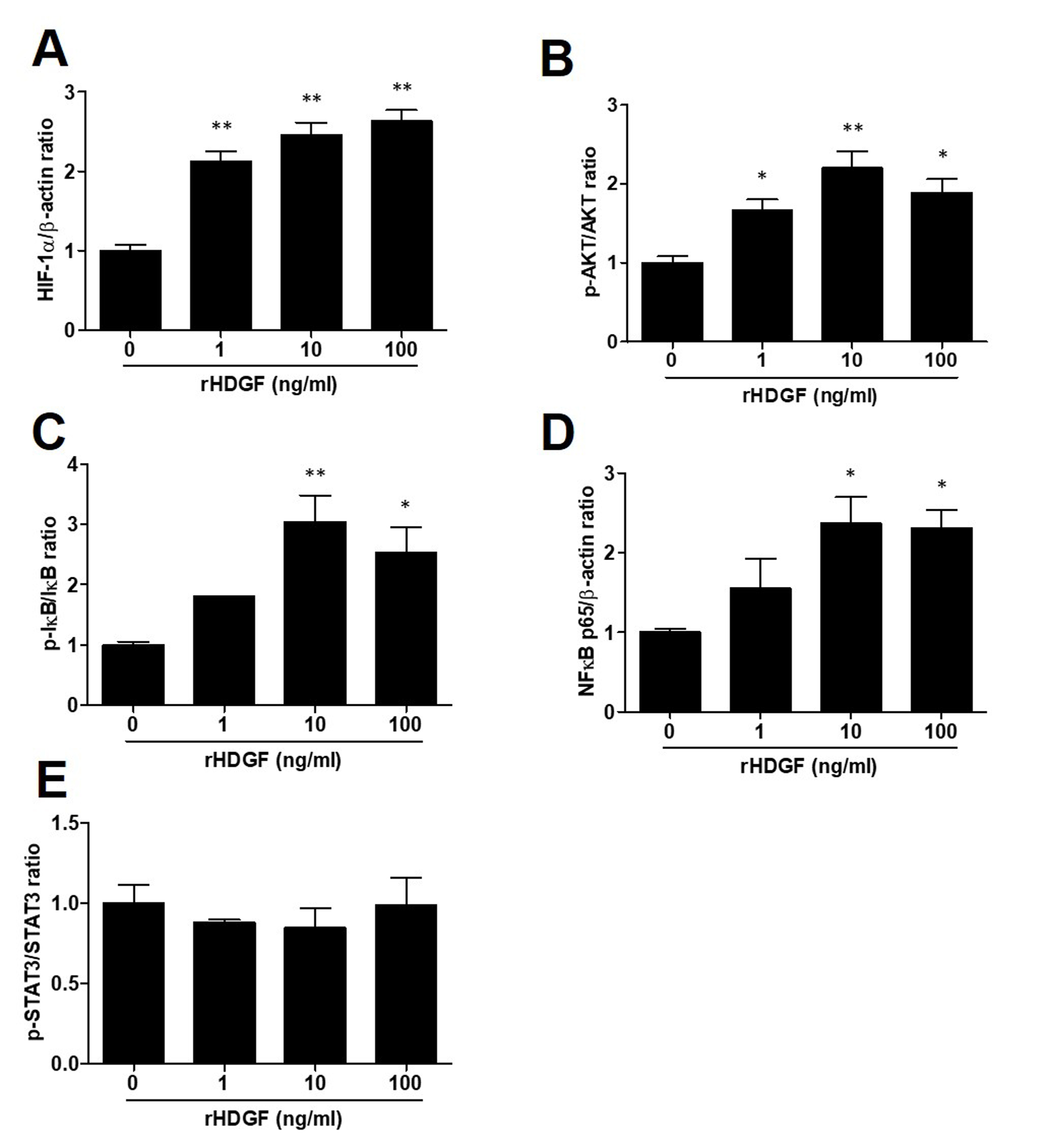
**

**Figure S3. HDGF triggered AKT/HIF-1α/NF-κB signaling in SCC4 oral cancer cells.** (A-E) Cells were treated with recombinant HDGF (1-100 ng/ml) for 24 h and then harvested for total protein extraction. The cell lysates were separated by SDS-PAGE and detected by Western blotting with the indicated primary antibodies, and the indicated protein levels were measured and quantified. β-Actin was used as an internal control for loading and transfer. Data were mean of three experiments. *, P<0.05; **, P<0.01; ns, not statistically significant.

**Figure S4. The Neutralizing antibody against nucleolin eliminates HDGF-stimulated AKT/HIF-1α/NF-κB/VEGF signaling in SCC4 oral cancer cells.** (A-E) SCC4 cells were treated with recombinant HDGF protein (100 ng/ml) in the presence of anti-NCL or anti-IgG antibody (5 μg/ml) for 24 h before total protein extraction. Cell lysates were subjected to Western blotting with the indicated antibodies, and the indicated protein levels were measured and quantified. β-Actin was used as an internal control for loading and transfer. Data were mean of three experiments. *, P<0.05; **, P<0.01; ns, not statistically significant.
